# Proper Microtubule Structure Is Vital for Timely Progression through Meiosis in Fission Yeast

**DOI:** 10.1371/journal.pone.0065082

**Published:** 2013-06-05

**Authors:** Akira Yamashita, Yoshihiro Fujita, Masayuki Yamamoto

**Affiliations:** 1 Laboratory of Gene Function, Kazusa DNA Research Institute, Kisarazu, Chiba, Japan; 2 Department of Biophysics and Biochemistry, Graduate School of Science, University of Tokyo, Hongo, Tokyo, Japan; University of Cambridge, United Kingdom

## Abstract

Cells of the fission yeast *Schizosaccharomyces pombe* normally reproduce by mitotic division in the haploid state. When subjected to nutrient starvation, two haploid cells fuse and undergo karyogamy, forming a diploid cell that initiates meiosis to form four haploid spores. Here, we show that deletion of the *mal3* gene, which encodes a homolog of microtubule regulator EB1, produces aberrant asci carrying more than four spores. The *mal3* deletion mutant cells have a disordered cytoplasmic microtubule structure during karyogamy and initiate meiosis before completion of karyogamy, resulting in twin haploid meiosis in the zygote. Treatment with anti-microtubule drugs mimics this phenotype. Mutants defective in karyogamy or mutants prone to initiate haploid meiosis exaggerate the phenotype of the *mal3* deletion mutant. Our results indicate that proper microtubule structure is required for ordered progression through the meiotic cycle. Furthermore, the results of our study suggest that fission yeast do not monitor ploidy during meiosis.

## Introduction

Karyogamy, a process in which two haploid nuclei fuse to produce a diploid nucleus, must occur prior to initiation of meiosis in haploid organisms such as the fission yeast *Schizosaccharomyces pombe*. Haploid fission yeast cells display one of two mating types, designated as *h^+^* and *h^−^.* Homothallic strains, designated as *h^90^*, undergo frequent switching of mating type between *h^+^* and *h^−^*, whereas heterothallic strains are fixed as either *h^+^* or *h^−^*. When starved for nutrients, particularly nitrogen, haploid cells conjugate with cells of the opposite mating type, after which they undergo karyogamy and form diploid zygotes. In conjugated diploid cells, expression of the *mei3* gene is induced [1], [2]. The *mei3* gene encodes an inhibitor of Pat1 kinase [3]. Pat1 kinase negatively regulates the initiation of meiosis [4], [5]. Once Pat1 is inactivated by Mei3, dephosphorylated Mei2, which is a critical target of Pat1, accumulates and triggers the initiation of meiosis [6]–[8].

Regulation of the temporal order of conjugation, karyogamy, and the initiation of meiosis is crucial for the generation of proper haploid spores. However, several mutants are known to undergo haploid meiosis in the absence of conjugation. For instance, temperature-sensitive *pat1* mutants initiate haploid meiosis at elevated temperatures [4], [5]. Expression of an activated form of Mei2 that has alanine substitutions at Pat1 target sites also induces haploid meiosis [6]. It is also known that *h^+^*/*h^+^* and *h^−/^h^−^* diploid cells can mate, and the resulting tetraploid cells occasionally produce more than four spores [9]. These spores are derived from “twin meiosis”, in that two nuclei initiate meiosis separately in the absence of karyogamy [9]. This suggests that fission yeast might not monitor the completion of karyogamy prior to undergoing meiosis. Here, we report that twin meiosis occurs even in diploid zygotes. We examined the meiotic phenotype of cells lacking the *mal3* gene that encodes a homolog of EB1, a well-characterized microtubule plus end-tracking protein originally identified as a binding partner of the tumor suppressor protein APC [10]. Mal3 is shown to be a crucial microtubule regulator, both in interphase and during mitotic division [11]–[13]. *mal3*Δ cells are characterized by a defective microtubule cytoskeleton, which causes aberrant cell morphology, and by defects in chromosome stability. In the present study, we investigated the meiotic phenotype of the *mal3*Δ strain, and found that correct cytoplasmic microtubule organization is crucial for proper meiotic progression.

## Materials and Methods

### Fission Yeast Strains, Genetic Analysis, and Growth Media

**Figure 1:**
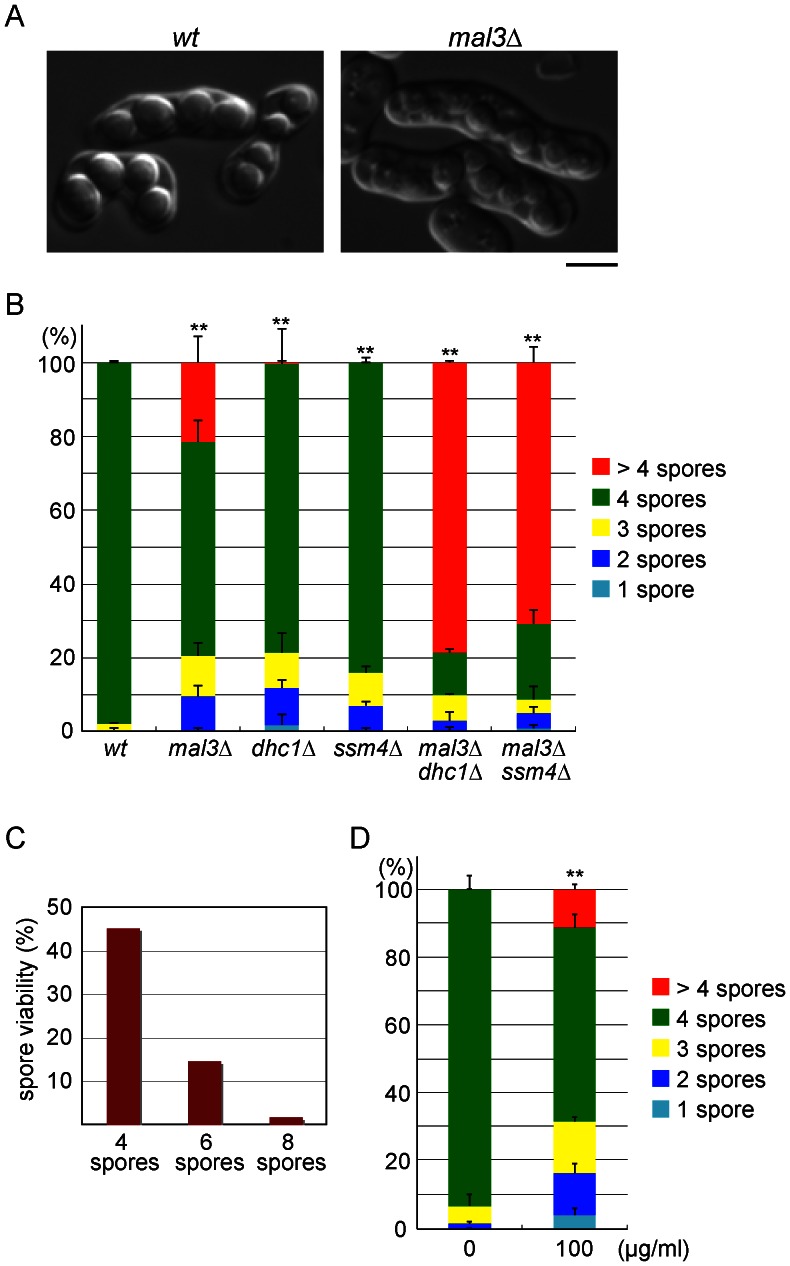
The *mal3*Δ mutant generates aberrant asci containing more than four spores. (**A**) Micrographs of spore-containing asci. Right panel shows asci produced by *mal3*Δ mutant cells, each carrying eight spores. Wild-type (JY450) and *mal3*Δ (JW794) homothallic haploid cells were grown on SSA sporulation medium at 30°C for 4 days to induce conjugation, meiosis, and sporulation. Bar = 5 µm. (**B**) Percentage of asci containing 1, 2, 3, 4, or >4 spores. Wild-type (JY450), *mal3*Δ (JW794), *dhc1*Δ (JW327), *ssm4*Δ (JW652), *mal3*Δ *dhc1*Δ (JW866), and *mal3*Δ *ssm4*Δ (J853) strains were induced to sporulate by incubating cells for 3 days on SSA medium at 30°C. More than 200 asci were examined for each strain. Error bars indicate standard deviations from three independent experiments. Asterisks indicate significant difference from the wild-type strain (**p<0.01 (Chi-square test)). (**C**) Viability of spores of the *mal3*Δ strain (JW794) from asci carrying 4, 6, or 8 spores. More than 80 spores from each ascus were dissected using a micromanipulator and incubated on rich YE medium at 30°C. (**D**) Percentage of asci containing 1, 2, 3, 4, or >4 spores generated by the wild-type strain (JY450) on medium containing the anti-microtubule drug thiabendazole. Sporulation was induced by cells at 30°C on SPA medium with or without 100 µg/mL thiabendazole. After incubation for 1 day, more than 200 asci were examined. Error bars indicate standard deviations from three independent experiments. Asterisks indicate significant difference from the wild-type strain (**p<0.01 (Chi-square test)).

Table 1 lists the *S. pombe* strains used in this study. General genetic analyses of the *S. pombe* strains followed previously described procedures [14]. Growth media used in the study included complete YE, minimal SD and MM [15], synthetic sporulation SSA [16], and sporulation SPA [14]. A standard protocol was used for gene tagging and deletion [17]. To determine percentage of asci containing 1, 2, 3, 4 or >4 spores, more than 200 asci were examined after incubation on SSA for 3 days (Fig. 1A and 3C) or on SPA for 1 day (Fig. 1D). Standard deviations were calculated from three independent experiments.

**Table 1:** *Schizosaccharomyces pombe* strains used in this study.

Strain	Genotype
JT165	*h^90^ mal3::kanR pat1-114 adde6-M216 leu1*
JT935	*h^90^ mal3::ura4^+^ GFP-atb2-kanR cut11-3mRFP-hphR ade6-M210 leu1 ura4-D18*
JT936	*h^90^ GFP-atb2-kanR cut11-3mRFP-hphR ade6-M210 leu1 ura4-D18*
JT937	*h^90^ mal3::ura4^+^ mei3-GFP-kanR CFP-atb2-natR cut11-3mRFP-hphR ade6-M216 leu1 ura4-D18*
JT939	*h^90^ mei3-GFP-kanR CFP-atb2-natR cut11-3mRFP-hphR ade6-M216 leu1 ura4-D18*
JW327	*h^90^ dhc1::ura4^+^ ade6-M216 leu1 ura4-D18*
JW652	*h^90^ ssm4::kanR ade6-M216 leu1*
JW794	*h^90^ mal3::kanR ade6-M216 leu1*
JW853	*h^90^ mal3::kanR ssm4::kanR ade6-M216 leu1*
JW866	*h^90^ mal3::kanR dhc1::ura4^+^ ade6-M216 leu1*
JY450	*h^90^ ade6-216 leu1*

### Live Cell Imaging

To induce meiosis, each strain was grown to mid-log phase in MM medium at 30°C, after which cells were collected, washed, and spotted onto plates containing SPA medium. After incubation for 4–6 hours at 30°C, cells were observed using the PersonalDV microscopy imaging system (Applied Precision, Issaquah, WA, USA) or under a fluorescence microscope (AxioPlan 2; Carl Zeiss, Oberkochen, Baden-Württemberg, Germany) equipped with a chilled CCD camera (CoolSNAP HQ2; PHOTOMETRICS, Tucson, AZ, USA) and MetaMorph software (Molecular Devices, Sunnyvale, CA, USA). To determine percentage of zyogotes containing one or two Mei3-GFP-positive nuclei, more than 200 cells were examined after incubation on SPA. Standard deviations were calculated from three independent experiments.

## Results

### Mal3 is Essential for Proper Spore Production

The sexual differentiation pathway, which involves mating, meiosis, and sporulation, was induced in *mal3*Δ cells in order to elucidate the role of Mal3 in meiosis. The mating and sporulation frequencies of the *mal3*Δ strain were comparable to those of the wild type; however, 21% of the *mal3*Δ cells generated aberrant asci containing more than four (at most eight) spores, a phenomenon that was observed infrequently in wild-type cells (Figs. 1A and 1B). We then determined the viability of spores in the aberrant asci produced by *mal3*Δ cells, and found that spore viability declined as the number of spores per ascus increased (45%, 15% and 2% in four-, six- and eight-spore asci, respectively, Fig. 1C). These results suggest that Mal3 plays a significant role in meiosis. Zygotes carrying less than three spores were rarely detected in the wild-type strain, but were observed more frequently in the *mal3*Δ strain (0.4% in *wt* versus 9.5% in *mal3*Δ, Fig. 1B). The higher frequency of zygotes carrying less than three spores could be due to a deficiency in microtubule-dependent nuclear movement during meiotic prophase, as has been shown to occur in dynein-dynactin mutants [18]–[20].

Mal3 is a crucial microtubule regulator, and consequently disruption of the *mal3* gene compromises the structure of microtubules [11]. We therefore examined whether impairment of the microtubule structure leads to the aberrant spore formation. In wild-type cells cultured in the presence of the microtubule-destabilizing drug thiabendazole, 11% of the spore-containing zygotes contained more than four spores (Fig. 1D), suggesting that disorder in the structure of microtubules is responsible for the extra-spore phenotype of the *mal3*Δ strain. Consistent with this observation, the *mto1*Δ strain, in which cytoplasmic microtubule nucleation is abolished [21]–[23], also showed the extra-spore phenotype (data not shown).

### Meiosis Proceeds without Karyogamy in *mal3*Δ Cells

**Figure 2:**
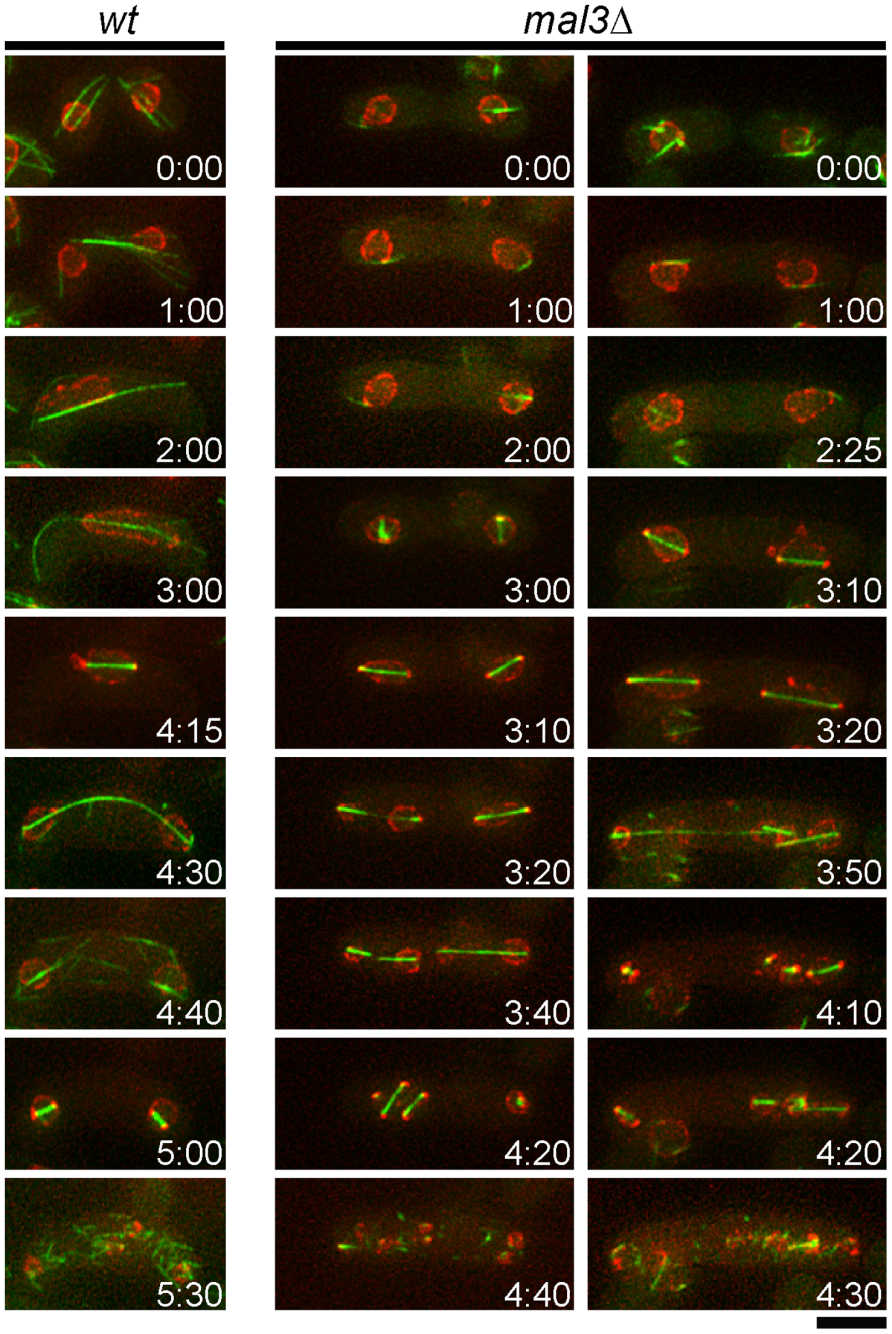
The *mal3*Δ strain initiates meiosis I before karyogamy is completed. Fluorescence micrographs of homothallic haploid cells of the wild-type (JT936) and *mal3*Δ (JT935) strains carrying the microtubule marker *GFP-atb2* (green) and nuclear envelope marker *cut11-3mRFP* (red). Cells were subjected to nitrogen starvation to induce conjugation and meiosis. Serial images taken at indicated times (hr:min) are shown for each zygote. Bar = 5 µm.

We next investigated how the extra-spore phenotype is elicited in the *mal3*Δ strain. Time-lapse observations made throughout the entire meiotic process, from mating to meiosis II, showed that meiotic divisions occurred before the completion of karyogamy in *mal3*Δ cells (Fig. 2). We utilized *mal3^+^* and *mal3*Δ strains carrying the *GFP-atb2* fusion gene encoding GFP-tubulin and the *cut11-3mRFP* gene encoding a fusion protein consisting of the nuclear envelope marker Cut11 [24] and three copies of mRFP. In wild-type cells, karyogamy occurred prior to meiosis I (Fig. 2, *wt*). In *mal3*Δ cells, cytoplasmic microtubules, which in wild-type cells bridge two approaching nuclei, were severely impaired. During karyogamy in budding yeast, nuclear congression is driven by microtubule dynamics [25], [26]. Disruption of the *BIM1* gene, which encodes the EB1 homolog in budding yeast, has been shown to lead to a defect in karyogamy [27]. We found that nuclear congression was defective in *mal3*Δ cells, as in *bim1* mutant budding yeast. In addition, *mal3*Δ cells entered meiosis I in the absence of karyogamy, leading to twin haploid meiosis in single zygotes. These observations suggest that formation of extra spores in *mal3*Δ cells is caused by the skipping of karyogamy.

We hypothesized that the delay in karyogamy might exacerbate the extra-spore phenotype of *mal3*Δ cells. To test this hypothesis, we constructed a mutant strain with deletions of both *mal3* and *dhc1*, which encodes the dynein heavy chain, and a mutant strain with deletions of both *mal3* and *ssm4*, which encodes the p150 glued subunit of the dynactin complex, since nuclear fusion is reportedly delayed in mutants lacking the dynein heavy chain [18]. These double mutant strains showed the exaggerated phenotype, as expected (79% in *mal3*Δ *dhc1*Δ and 71% in *mal3*Δ *ssm4*Δ versus 21% in *mal3*Δ, Fig. 1B).

### Twin Haploid Meiosis Occurs in *mal3*Δ Cells

**Figure 3:**
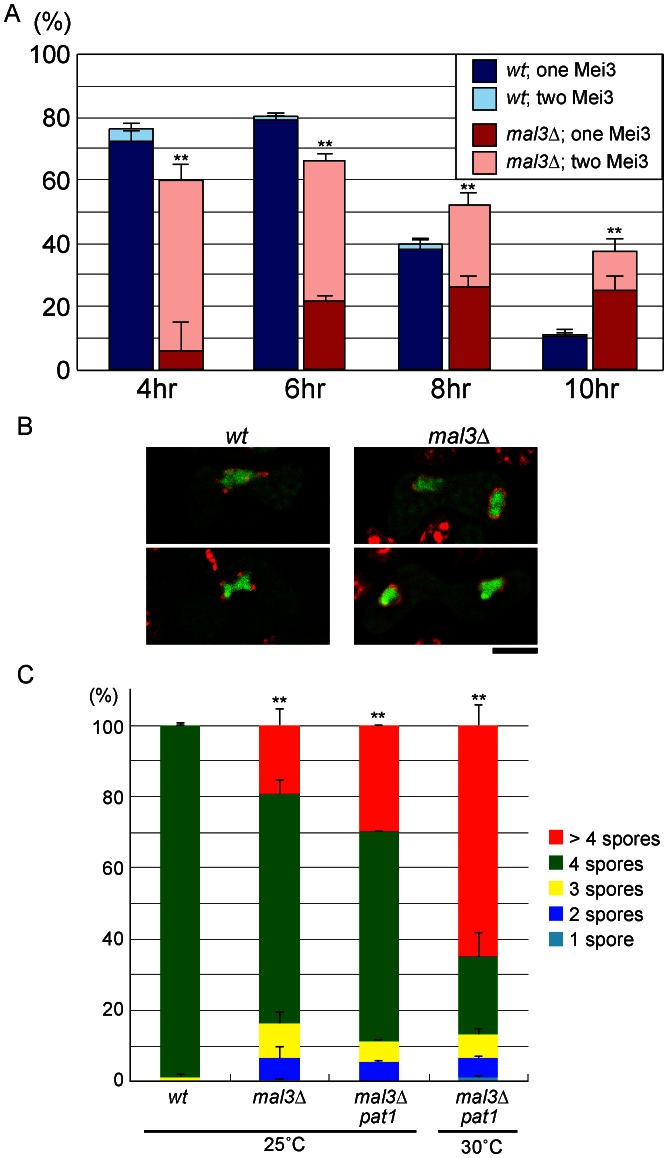
Twin haploid meiosis proceeds in a single *mal3*Δ zygote. (**A**) Percentage of *mal3*Δ zygotes containing one or two nuclei expressing Mei3-GFP. Wild-type (JT939) and *mal3*Δ (JT937) homothallic haploid cells carrying *mei3-GFP* and *cut11-3mRFP* were subjected to nitrogen starvation to induce conjugation and meiosis. More than 200 cells were examined microscopically following incubation for the indicated times to determine the number of zygotes expressing Mei3-GFP. Error bars indicate standard deviations from three independent experiments. Asterisks indicate significant difference from the wild-type strain (**p<0.01 (Chi-square test)). (**B**) Fluorescent micrographs of typical zygotes expressing Mei3-GFP in two nuclei. Wild-type (JT939) and *mal3*Δ (JT937) homothallic haploid cells were starved for nitrogen as in (A). Mei3-GFP is shown in green and the nuclear membrane marker Cut11-3mRFP is shown in red. Bar = 5 µm. (**C**) Percentage of asci containing 1, 2, 3, 4, or >4 spores generated by cells of the wild-type (JY450), *mal3*Δ (JW794), and *mal3*Δ *pat1* (JT165) strains. Sporulation was induced in each strain by growth for 3 days on SSA medium at either 25°C or 30°C. More than 200 asci were examined for each strain. Error bars indicate standard deviations from three independent experiments. Asterisks indicate significant difference from the wild-type strain (**p<0.01 (Chi-square test)).

Next, we monitored meiotic initiation by following the expression of Mei3, which is expressed only in cells stimulated to initiate meiosis as a result of nutritional starvation and heterozygosity [1]. Expression of Mei3-GFP was consistently observed in the fused nucleus of most nitrogen-starved wild-type cells (Figs. 3A and 3B). In clear contrast, the frequency of cells containing two Mei3-GFP-positive nuclei was higher in the *mal3*Δ mutant. This observation supports the idea that two haploid meioses occur prematurely in the *mal3*Δ strain. We then examined the effect produced by accelerating the initiation of meiosis by inhibiting the activity of the Pat1 kinase in *mal3*Δ cells. Pat1 kinase is inhibited by Mei3, thereby triggering meiosis [3]. Introduction of a temperature sensitive mutation in the *pat1* gene resulted in exaggerated extra-spore formation in *mal3*Δ cells cultured at the semi-permissive temperature of the *pat1* mutation (Fig. 3C, 30°C).

## Discussion

In the present study, we demonstrated that fission yeast can initiate meiosis before the completion of karyogamy. Nuclear migration during karyogamy depends entirely upon cytoplasmic microtubules. It has been reported that cytoplasmic dynein plays a role in karyogamy, although nuclear fusion is eventually accomplished even in dynein deletion mutants [18]. In the budding yeast *Saccharomyces cerevisiae*, cytoplasmic microtubule plus-end interactions and depolymerization dependent upon the minus-end-directed kinesin Kar3 is thought to generate forces that drive nuclear congression during karyogamy [25]. Recently, it has been shown that Kar3 anchored at a spindle pole body (SPB) could exert the pulling forces on microtubules nucleated from the SPB of the mating partner nucleus and drive nuclear migration [26]. In fission yeast, zygotes lacking both *dhc1*, which encodes dynein heavy chain, and *klp2*, which encodes a kinesin-like protein homologous to Kar3, form asci containing more than four spores at a high frequency, suggesting that Klp2 kinesin is involved in karyogamy [28]. An intriguing question for further investigation centers upon how dynein and kinesin are coordinated in this process.

Our results show that even spores that are produced by “twin haploid meiosis” retain viability, although at a very low level (Fig. 1C). One potential reason that *S. pombe* has not developed checkpoint mechanisms to monitor the completion of karyogamy is that it has only three chromosomes. Due to the relatively small number of chromosomes, the complete genome could occasionally be passed to spores produced through haploid meiosis. *Schizosaccharomyces japonicus*, a species closely related to *S. pombe*, generates eight spores in each ascus through post-meiotic nuclear division [29], [30], which is distinct from our findings in *S. pombe* mutant zygotes. The meiotic and sporulation phenotypes of karyogamy-defective *S. japonicus* mutants remain elusive. Comparative analyses of the two related species might provide significant information regarding regulation of the initiation and progression of meiosis.
